# A feasibility study of pre-sleep audio and visual alpha brain entrainment for people with chronic pain and sleep disturbance

**DOI:** 10.3389/fpain.2023.1096084

**Published:** 2023-02-23

**Authors:** Stephen J. Halpin, Alexander J. Casson, Nicole K. Y. Tang, Anthony K. P. Jones, Rory J. O’Connor, Manoj Sivan

**Affiliations:** ^1^ Academic Department of Rehabilitation Medicine, Leeds Institute of Rheumatic and Musculoskeletal Medicine, University of Leeds, Leeds, United Kingdom; ^2^ Human Pain Research Group, Division of Neuroscience and Experimental Psychology, University of Manchester, Manchester, United Kingdom; ^3^ Department of Electrical and Electronic Engineering, University of Manchester, Manchester, United Kingdom; ^4^ Department of Psychology, University of Warwick, Warwick, United Kingdom

**Keywords:** brain stimulation, neuromodulation, visual, binaural beats, smartphone, fatigue, mood

## Abstract

**Introduction:**

Chronic pain and sleep disturbance are bi-directionally related. Cortical electrical activity in the alpha frequency band can be enhanced with sensory stimulation *via* the phenomenon of entrainment, and may reduce pain perception. A smartphone based programme which delivers 10 Hz stimulation through flickering light or binaural beats was developed for use at night, pre-sleep, with the aim of improving night time pain and sleep and thereby subsequent pain and related daytime symptoms. The aim of this study was to assess the feasibility and give an indication of effect of this programme for individuals with chronic pain and sleep disturbance.

**Materials and methods:**

In a non-controlled feasibility study participants used audio or visual alpha entrainment for 30 min pre-sleep each night for 4 weeks, following a 1 week baseline period. The study was pre-registered at ClinicalTrials.gov with the ID NCT04176861.

**Results:**

28 participants (79% female, mean age 45 years) completed the study with high levels of data completeness (86%) and intervention adherence (92%). Daily sleep diaries showed an increase compared to baseline in total sleep time of 29 min (*p* = 0.0033), reduction in sleep onset latency of 13 min (*p* = 0.0043), and increase in sleep efficiency of 4.7% (*p* = 0.0009). Daily 0–10 numerical rating scale of average pain at night improved by 0.5 points compared to baseline (*p* = 0.027). Standardised questionnaires showed significant within-participant improvements in sleep quality (change in median Global PSQI from 16 to 12.5), pain interference (change in median BPI Pain Interference from 7.5 to 6.8), fatigue (change in median MFI total score from 82.5 to 77), and depression and anxiety (change in median HADS depression score from 12 to 10.5 and anxiety from 13.5 to 11).

**Discussion:**

Pre-sleep use of a smartphone programme for alpha entrainment by audio or visual stimulation was feasible for individuals with chronic pain and sleep disturbance. The effect on symptoms requires further exploration in controlled studies.

## 1. Introduction

Chronic pain represents a significant unmet global health need. It is highly prevalent, affecting one-fifth to one-third of adults (1, 2) and represents one of the greatest contributors to disability globally (3). Conventional analgesic medications have poor efficacy and unfavourable side effect profiles when used for chronic pain (4, 5) and many individuals regard their pain as inadequately controlled (6). New paradigms of treatment are required, which is why the interplay between sleep and chronic pain has received increased attention in recent years as a priority area for research (7, 8).

Sleep problems are very common in people living with chronic painful conditions (9, 10), and almost universal in those with fibromyalgia (11). The relationship between pain and sleep is bidirectional (12), and is seen to operate on both short (13) and long (14, 15) time scales. Observationally, non-restorative sleep has been found to be a strong independent predictor of new onset widespread pain (16), and experimental sleep deprivation and sleep fragmentation increases pain (17). There is a strong rationale for linking novel approaches to the intractable problem of chronic pain with sleep disturbance, given this close relationship.

Alpha entrainment is a neuromodulatory approach that has the potential to help people living with chronic pain. Entrainment is when an oscillating system becomes synchronised in phase to an external periodic force. Cortical electrical oscillations, or “brainwaves”, demonstrate this phenomenon in response to rhythmic stimuli, which can be sensory or direct electric or magnetic stimulation (18). Alpha entrainment refers to modulation of cortical activity in the alpha band (8–12 Hz). The overall role of the alpha rhythm has been proposed as providing inhibitory gating between brain regions (19), thereby re-routing resources and information to task-relevant areas. It is involved in pain expectation (20), attention to pain (21) and expectation of pain relief (22) and therefore provides a promising avenue for novel treatments, considering its entrainment is technologically achievable through non-invasive sensory stimulation. Alpha entrainment has been found to reduce experimental laser-induced pain in healthy participants (23) including with the electrophysiological correlate of reduced amplitude of the laser evoked potential (24). In laboratory studies with individuals with chronic musculoskeletal pain, 4 min of 10 Hz sensory stimulation has been shown to successfully entrain alpha and decrease pain (25) and the degree of frontal alpha power increase is found to correlate with the reduction in pain (moderate strength correlation, Pearson *r* 0.33 for pain intensity, 0.40 for pain unpleasantness) (26). To our knowledge alpha entrainment has not previously been used pre-sleep in those with chronic pain, but other forms of non-invasive brainwave entrainment have been investigated for use at night in the home environment in this group. Audio-visual stimulation decreasing in frequency from 8 to 2 Hz was used by participants with chronic pain and insomnia aiming to aid sleep onset. It was shown that entrainment to the stimuli (in this case delta power) was successful (27), and in both younger (28) and older (29) participants who had chronic pain and insomnia improvements were seen in both symptoms. In a pilot randomised controlled trial, adherence to the intervention was 99% and participants found it easy to use (30), indicating that the concept of audio-visual stimulation at night may be acceptable in this population. Outside of chronic pain, alpha entrainment has been used by healthy participants as one part of a stimulation programme aiming to optimise sleep quality (31). In this pilot trial, the first quarter of a 90 min sensory stimulation programme used 8 Hz (lower range of the alpha frequency) stimulation, and subsequently lower frequencies, and found improvements in participant reported sleep quality. Alpha entrainment therefore holds promise for the treatment of the closely linked problems of chronic pain and sleep disturbance. We designed a smartphone application called home-based Brainwave Entrainment Technology (hBET) to deliver 10 Hz alpha stimulation. This is the first time this modality has been used pre-sleep by those with chronic pain.

The aim of this study is to explore the feasibility of pre-sleep use of hBET with individuals with chronic pain and sleep disturbance and give an indication of the effect of the treatment.

## 2. Materials and methods

This was an uncontrolled feasibility study, comparing pre- and post-intervention measures. There was no randomisation and participants were not blinded to the intervention received. Participants were 28 individuals living with chronic pain and sleep disturbance, recruited from NHS clinics dealing with chronic pain in two regions in the north of England (Leeds and Manchester) and *via* online publicity materials. Inclusion criteria were: age 18–80, non-cancer pain of over 3 months' duration including nocturnal pain of at least 4/10 (on 0–10 NRS), and self-reported sleep difﬁculties (defined as trouble falling asleep, difﬁculty staying asleep, waking up too early, or waking up unrefreshed on 3 or more nights per week during the past month). Individuals were excluded from participating if they had: any seizure disorder, photosensitivity, planned pain intervention during the study period, hearing and vision problems causing inability to use the stimulation, or inability to consent.

### 2.1. Intervention

**Figure 1:**
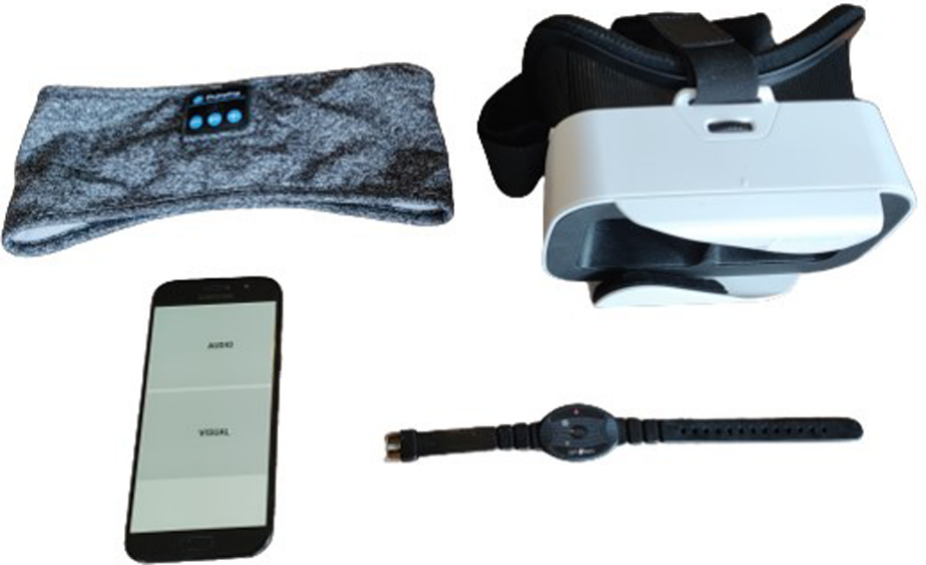
Equipment used; sleep headband with integrated wireless headphones, headset to hold phone for visual stimulation mode, smartphone with hBET app loaded, motionwatch 8 actigraph watch.

The hBET programme is a smartphone application specifically developed by the Human Pain Research Group (32) (a collaboration between researchers at the Universities of Manchester, Leeds and Liverpool, UK) to provide repetitive stimulation at 10 Hz by either visual or auditory modalities for investigation of the treatment of chronic pain. Development of the application (33) and user co-design (34) have been reported. The 10 Hz frequency was chosen as it is at the centre of the alpha band, and was found to more effectively reduce experimental pain than high (12 Hz) or low (8 Hz) alpha (23). This is an example of open-loop stimulation, as the programme feeds in 10 Hz stimulation with no reference to participants' online brainwave state or individualised peak alpha (35). The visual programme uses the smartphone screen to create 10 Hz visual flicker by alternating between white and black screen at this frequency. A virtual reality headset is used to hold the phone in front of participants' eyes and exclude external light sources. Participants have their eyes closed during the stimulation. The screen brightness is pre-set at mid-range, but is under participants' control. The auditory programme utilises binaural beats to create 10 Hz stimulation since a 10 Hz tone is below the range of human hearing. A binaural beat is produced when different tones are presented to each ear, with the binaural beat frequency being the difference between the two tones (36). Tones at 400 Hz and 410 Hz are used in hBET as this range has been shown to produce the binaural beat effect most strongly (37). It is therefore necessary that headphones are used rather than an external speaker. For increased comfort in a lying position, participants are provided with a sleep headband with integrated headphones [model PT28, Perytong, Shenzhen, China]. The volume of auditory stimulation is under participants' control. The equipment participants used in the study is shown in Figure 1.

### 2.2. Procedures

Remote processes were used due to the Covid-19 pandemic. Participants were familiarised with the study equipment, sleep and pain diary and questionnaire schedule *via* an online videoconference meeting. There was a one-week baseline period followed by a four-week intervention period, during which time participants were asked to use hBET each evening. It was advised to be used immediately pre-sleep, when the participant was settled in bed and ready to try to get to sleep. The stimulation ceases after 30 min but could be restarted if desired. Participants had the choice to use the audio or visual option on any night, which allowed for the range of user preferences found on previous studies (34).

### 2.3. Measures

Demographic information and past medical history including pain diagnosis and medication use was collected from participants' own reports using a paper questionnaire at baseline. Diagnoses were not extracted from medical records or reconfirmed by the study team.

**Figure 2:**
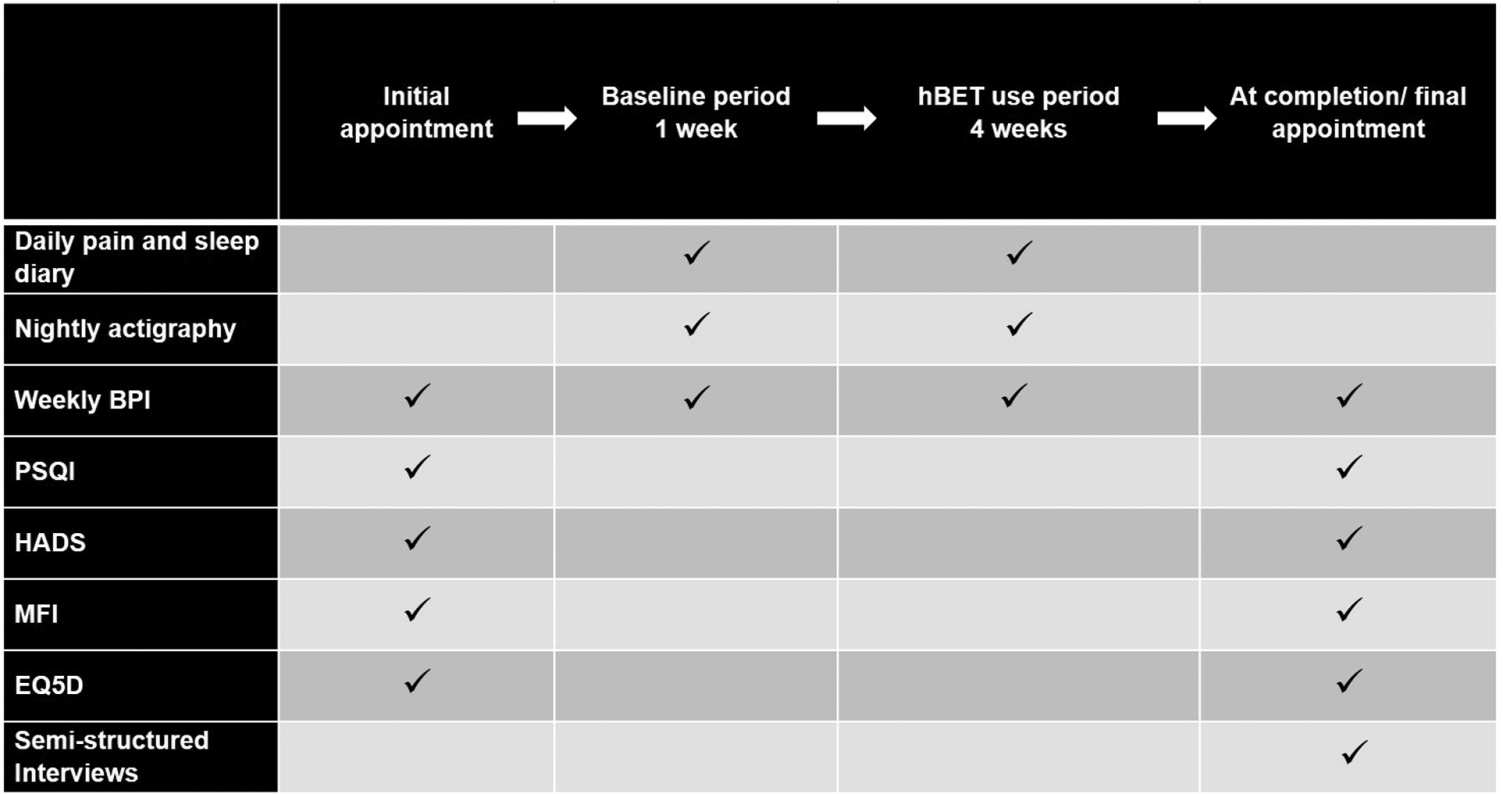
Study flow and assessment schedule.

A pain and sleep diary was completed each morning. This incorporated 0–10 numerical rating scale (NRS) for average pain over the last 24 h and average pain last night, and sleep timing used wording conforming to the consensus sleep diary (38). Sleep parameters calculated as follows: total sleep time is the time between trying to fall asleep to final awakening, minus the sleep latency and the total time awake after sleep onset; sleep efficiency is total sleep time divided by duration of the sleep episode, which is the time from starting to try to sleep to getting out of bed, (presented as a percentage). Sleep onset latency and Wake after sleep onset do not require calculations and are addressed directly on the sleep diary. In addition, in the diary participants rated the quality of their sleep and how refreshed they felt in the morning on a 0–5 NRS. Nightly actigraphy was also used to monitor sleep using Motionwatch 8 [CamNtech Ltd, Cambridge, UK]. Standardised questionnaires were the Brief Pain Inventory (39) completed weekly, the Pittsburgh Sleep Quality Index (40), Multidimensional Fatigue Inventory (41), Hospital Anxiety and Depression Scale (42), and five level EQ-5D (43), all completed at baseline and study completion. Qualitative data on acceptability and user experience were also gathered through semi-structured interviews with each participant at the completion of the study. These are presented elsewhere. A summary of the study flow and outcome measures schedule is provided in Figure 2.

### 2.4. Analysis

Data from questionnaires were scored using standardised methods for each measure. Sleep and pain diaries were converted to digital format (Microsoft Excel 2016) for the calculation of sleep parameters as described above.

Data from Motionwatches were downloaded and processed using the bespoke software Motionware (version 1.2.28, CamNtech, Cambridge, UK). Sleep periods for each night of data were marked up according to the watch marker button presses, which participants were instructed to press at the beginning and end of each sleep episode. Where these were absent the period was marked up manually based on triangulation of the actigraphy data and the sleep diary. In most cases the participant had omitted to press the marker button upon waking, and the end of the sleep episode could be reliably judged from the watch being taken off and this corresponding to the diary report of final rise time. When the sleep period could not confidently be marked up the data for that night were not included.

To explore the impact of the intervention, pre- and post- measures were compared on a within-participant basis, using the baseline week compared to periods when hBET was used. Following the baseline week, participants had the choice each night whether to use Audio or Visual hBET. Since each option is a strategy to achieve the same effect of alpha entrainment, the primary analysis considers hBET as one intervention irrespective of modality. This allows an element of personalisation in the intervention to account for individual preference and improve the likelihood of engagement. Disaggregated results for Audio and Visual are presented as Supplementary Material. Data from each condition (visual and audio) were included in the analysis if the participant used the condition for at least 5 nights, to account for periods where participants trialled a modality but had a strong user preference for the other modality.

Statistical analyses were conducted using paired *t*-tests or Wilcoxon sign rank tests for data which were non-normally distributed. Effect sizes were calculated with Cohen's *d* or, in the case of non-normally distributed data, the effect size r was calculated by dividing the Z-statistic from the Wilcoxon sign rank test by the square root of the sample size. Cohen's *d* was considered small if 0.2–0.5, medium if 0.5–0.8, and large if >0.8. Effect size r was considered small if 0.1–0.3, moderate if 0.3–0.5, large if >0.5 (44).

Ethical and regulatory approval for the study was granted by the Health Research Authority and NHS Research Ethics Committee reference 19/YH/0313 and procedures followed were in accordance with the Helsinki Declaration. The study was pre-registered at ClinicalTrials.gov with the ID NCT04176861.

## 3. Results

Twenty eight individuals (22 female, 6 male) participated and their demographic and background data are shown in Table 1.

**Table 1:** Participant background and demographics (*n* = 28).

Female	22 (79%)
Age in years (mean, SD)	45 (12)
Employment status
Unemployed	15 (54%)
Full time work	6 (21%)
Part time work	3 (11%)
Retired	4 (14%)
Duration of pain in years (median, range)	9 (1.2–40)
Duration of sleep problems in years (median, range)	8 (1.2–30)
Age in years at pain onset (median, range)	33 (13–69)
Diagnosis (reported by participant)
Fibromyalgia/chronic widespread pain syndrome	26 (93%)
Osteoarthritis	8 (29%)
Chronic low back pain	4 (14%)
Chronic fatigue syndrome	4 (14%)
Migraine/cluster headaches	2 (7%)
Trigeminal neuralgia	1 (4%)
Complex regional pain syndrome	1 (4%)
Medication use
Number of pain medications (median, range)	2 (0–5)
Opioid	16 (57%)
Gabapentinoid	14 (50%)
Paracetamol	14 (50%)
Non-steroidal anti-inflammatory	7 (25%)
Tricyclic antidepressant	6 (21%)
Serotonin and noradrenaline reuptake inhibitor	4 (14%)
Benzodiazepine	3 (11%)
Triptan	2 (7%)
Other	2 (7%)

**Figure 3:**
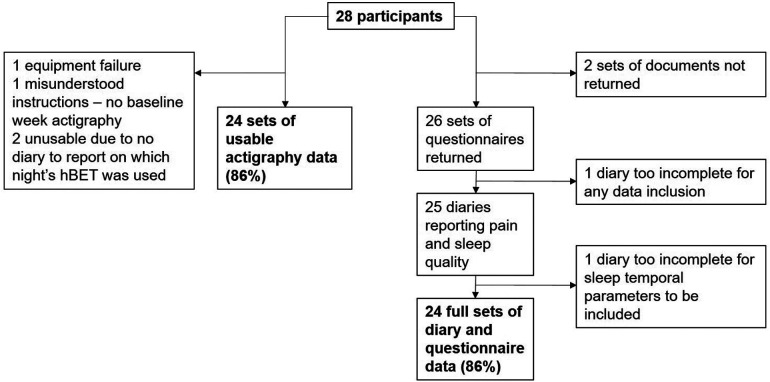
Data completeness and sources of attrition.

To inform the feasibility assessment, data completeness and attrition are displayed in Figure 3.

Overall, participants chose to use Audio hBET on 458 nights and Visual hBET on 187 nights. This gives an adherence rate of 92% in participants for whom diary records were available (hBET used on 645 of 700 available nights). Five participants tried Visual hBET for fewer than 5 nights before choosing to discontinue it and return to Audio. These quickly aborted periods of Visual use are not included in the analysis, leaving 175 nights of Visual hBET use.

### 3.1. Sleep and pain diaries

Sleep and pain diary data for hBET use periods compared to baseline periods are shown in Table 2. There was a small improvement in average pain scores both at night and over 24 h, as reported on the 0–10 numerical rating scale (NRS), and this difference was statistically significant but small in magnitude when averaged over all participants (effect size small; 0.47 and 0.41 respectively). Larger effect sizes were seen in the improvements in total sleep time and sleep efficiency (medium effect size; 0.67 and 0.78 respectively). Ratings given each morning on the quality of sleep did not improve but ratings of how refreshed participants felt did improve from a median of 2 to 2.75 on a 0–5 NRS.

**Table 2:** Sleep diary results.

	*n*	Mean (SD) at baseline (175 nights)	Mean (SD) in hBET condition (633 nights)	Change (hBET compared to baseline)	*p*-value for paired difference^a^ between hBET and baseline	Effect size
Average pain over 24 h (0–10 NRS)	25	6.4 (1.9)	6.1 (1.8)	−0.3 (0.7)	**0** **.** **0499**	0.41^b^
Average pain at night (0–10 NRS)	25	5.9 (2.0)	5.4 (2.1)	−0.5 (1.1)	**0**.**0265**	0.47^b^
	** **	**Mean (SD) at Baseline (168 nights)**	**Mean (SD) in hBET condition (610 nights)**	**Change [mean (SD)]**	** **	** **
Sleep Onset Latency (mins)	24	51.0 (38.8)	38.4 (26.3)	−12.6 (23.7)	**0**.**0043**	0.58^c^
Wake After Sleep Onset (mins)	24	36.8 (32.6)	27.4 (26.7)	−9.0 (19.3)	**0**.**0333**	0.43^c^
Total Sleep Time (mins)	24	389.7 (80.4)	419.1 (78.8)	29.4 (43.9)	**0**.**0033**	0.67^b^
Sleep efficiency (%)	24	74.6 (11.1)	79.3 (11.1)	4.7 (6.1)	**0**.**0009**	0.78^b^
	** **	**Median (IQR) at Baseline**	**Median (IQR) in hBET condition**	**Change [median (IQR)]**	** **	** **
Median number of awakenings	24	3 (2)	2 (1)	−1 (1)	**0**.**0074**	0.55^c^
Median quality rating (0–5 scale)	24	3 (1)	3 (1)	0 (0.125)	0.4063	
Median refreshed rating (0–5 scale)	24	2 (1)	2.75 (1)	1 (1)	**0**.**0004**	0.72^c^

^a^ Paired *t*-test for continuous and normally distributed variables, Wilcoxon sign rank test for non-continuous or non-normally distributed variables.

^b^ Cohen's *d* for *t*-tests.

^c^ r for Wilcoxon sign rank tests.

Bold values indicates *P*<0.05.

### 3.2. Actigraphy

No significant difference in sleep was seen with hBET compared to baseline when measured with actigraphy, as shown in Table 3. Actigraphy results diverged from sleep diary results, particularly in Sleep Onset Latency, which was on average reported to be 28 min higher in diaries than estimated by actigraphy, and in Wake After Sleep Onset, which was reported to be 35 min lower in diaries than estimated by actigraphy.

**Table 3:** Actigraphy results (n = 24).

	Mean (SD) at Baseline (165 nights)	Mean (SD) in hBET condition (538 nights)	Change (hBET compared to baseline)	*p* value for paired difference^a^ between hBET and baseline
Sleep Onset Latency (mins)	20.2 (27.8)	18.9 (25.6)	−1.3 (12.9)	0.9317
Wake After Sleep Onset (mins)	67.8 (44.0)	63.7 (32.4)	−4.0 (18.0)	0.2831
Total Sleep Time (mins)	398.8 (85.6)	402.0 (83.5)	3.1 (38.3)	0.6926
Sleep efficiency (%)	81.2 (10.6)	82.2 (10.5)	1.0 (3.5)	0.3914

^a^ Paired *t*-test for continuous and normally distributed variables, Wilcoxon sign rank test for non-continuous or non-normally distributed variables.

### 3.3. Questionnaires

Standardised questionnaires conducted at baseline and completion demonstrate improvements in sleep quality, fatigue, mood and pain interference, as shown in Table 4. In addition, the Brief Pain Inventory has a specific question on the extent to which pain is felt to interfere with sleep, scored from 0 (does not interfere) to 10 (completely interferes). The median response fell from 7.8 at baseline to 6.8 at the end of the study (*p* = 0.004) and is of particular interest given the hypothesised mechanism of action of the intervention. (Note that this is distinct from the overall Pain Interference score, reported in Table 4).

**Table 4:** Questionnaire data (n = 26).

	Baseline median (IQR)	Completion^a^ median (IQR)	*p*-value^b^	Effect size (r)
Global PSQI	16 (4.5)	12.5 (8.25)	**0** **.** **0016**	0.62
MFI Total	82.5 (19.75)	77 (15.75)	**0**.**0089**	0.51
HADS Depression	12 (5.25)	10.5 (6.25)	**0**.**0095**	0.51
HADS Anxiety	13.5 (7.5)	11 (6.5)	**0**.**0107**	0.50
BPI Pain Severity	6.3 (2.6)	6.0 (2.8)	0.2711	
BPI Pain Interference	7.5 (3.3)	6.8 (3.1)	**0**.**004**	0.56
EQ-5D-5L index value	0.27 (0.41)	0.32 (0.48)	0.1711	

PSQI, pittsburgh sleep quality index; MFI, multidimensional fatigue inventory; HADS, hospital anxiety and depression scale, BPI, brief pain inventory; EQ-5D-5L, the 5 level EuroQol score.

^a^ In the case of BPI, this includes measures taken during hBET use, as well as on completion.

^b^ Wilcoxon sign rank test.

Bold values indicates *P*<0.05.

### 3.4. Responder analysis

An exploratory post-hoc responder analysis was conducted based on whether participants reported an improvement in the Brief Pain Inventory pain interference score by at least 1 point, which has been suggested as the minimal clinically important change (45). Partial response was defined as an improvement less than 1 point, and non-response being no change or worsening. This measure was selected on the basis of the hypothesis that hBET exerts is effect by improving pain and sleep, and consequently the overall impact and intrusiveness of this cluster of related symptoms, better captured in this compound metric than in a single item NRS such as pain severity. This is supported by users' descriptions of how sleep and pain interact, which involves mood and activity levels (expanded on in qualitative findings from this study, published elsewhere), which grounds this choice in the experience of users. The aim of this analysis was to explore the utility of this approach, rather than to make conclusions on treatment effect. “Responders”, as defined in this way, tended to have better improvements across the range of self-reported symptom areas and actigraphy, as summarised in Table 5.

**Table 5:** Responder analysis.

	All hBET	Responders *n* = 9	Partial responders *n* = 9	Non-responders *n* = 7
Pain diary – mean (SD)
Change in average pain over 24 h (0–10 NRS)	−0.3 (0.7)	−**0.5 (0.8)**	−**0.2** (**0.7)**	−**0.1** (**0.6)**
Change in average pain at night (0–10 NRS)	−0.5 (1.1)	−**0.9** (**1.2)**	−**0.4** (**1.2)**	−**0.1** (**0.5)**
Sleep Diary – mean (SD)
Change SOL (mins)	−12.6 (23.7)	−8.9 (12.3)	−14.6 (33.4)	−14.3 (21.7)
Change in mean WASO (mins)	−9.0 (19.3)	−**18.6** (**23.1)**	−2.3 (18.3)	−6.7 (12.7)
Change in mean TST (mins)	29.4 (43.9)	21.5 (21.7)	34.0 (59.3)	32.5 (44.9)
Change in sleep efficiency (%)	4.7 (6.1)	**5.3** (**6.6)**	**5.0** (**7.4)**	**3.8** (**4.1)**
Median quality rating (0–5 scale)	0 (0.125)	0.3 (0.4)	0.4 (0.5)	−0.4 (0.8)
Median refreshed rating (0–5 scale)	1 (1)	**1.1** (**0.2)**	**0.8** (**0.7)**	**0.4** (**0.9)**
Number of awakenings	−1 (1)	−**1.3** (**1.0)**	−**0.8** (**1.7)**	−**0.3** (**1.0)**
Change in % nights with quality rated 3+	8.7 (31.5)	**17.3** (**22.6)**	**10.5** (**25.5)**	−**9.6** (**28.9)**
Change in % mornings with refreshed rated 3+	19.6 (26.2)	34.0* (**18.8)**	**14.7** (**18.0)**	**9.5** (**15.8)**
Actigraphy - mean (SD)				
Change in mean SOL (mins)	−1.3 (12.9)	−**5.0** (**17.1)**	−**2.2** (**10.6)**	**2.0** (**10.4)**
Change in mean WASO (mins)	−4.0 (18.0)	−**10.1** (**25.2)**	−0.1 (9.7)	−5.2 (14.7)
Change in mean TST (mins)	3.1 (38.3)	−3.2 (35.9)	9.7 (32.8)	−7.0 (43.0)
Change in sleep efficiency (%)	1.0 (3.5)	**2.2** (**3.1)**	**1.2** (**3.5)**	−**0.5** (**4.2)**
Questionnaire data - median (IQR)
Change in Global PSQI score	−1.5 (4.25)	−3.0** (**6.0)**	0.0 (4.0)	−1.0 (2.0)
Change in MFI Total score	−3.5 (9.0)	−**7.0** (**3.0)**	−**2.0** (**13.0)**	**2.0** (**3.0)**
Change in HADS Depression score	−1 (4)	−1 (2.5)	0 (5)	−1 (4)
Change in HADS Anxiety score	−1 (3.25)	−1 (4)	−1 (3.5)	−2 (4)
Change in BPI Pain Interference with sleep	−0.8 (2.3)	−2.5** (**1.9)**	−**0.3** (**1.3)**	**0.0** (**1.8)**
Change in BPI Pain Severity	−0.1 (1.5)	−1.3** (**1.6)**	**0.0** (**0.5)**	**0.5** (**0.3)**
Change in EQ-5D-5L index value	0.0 (0.3)	0.25** (**0.35)**	**0.0** (**0.22)**	−**0.01** (**0.11)**
*Change in BPI Pain Interference*^a^	−*0.5* (*1.5)*	−*1.9* (*0.6)*	−*0.3* (*0.5)*	*0.4* (*0.7)*

Responders are participants who reported an improvement in the Brief Pain Inventory pain interference score by at least 1 point, partial responders by less than 1 point, non-responders showed no change or worsening.

^a^ This is the variable by which the responder categories were defined.

* Responder group significantly different to other groups combined (independent samples *T* test, *p* < 0.05).

** Responder group significantly different to other groups combined (Mann–Whitney *U*-test, *p* < 0.05).

Bold values indicates other outcomes which improve most in responders.

**Figure 4:**
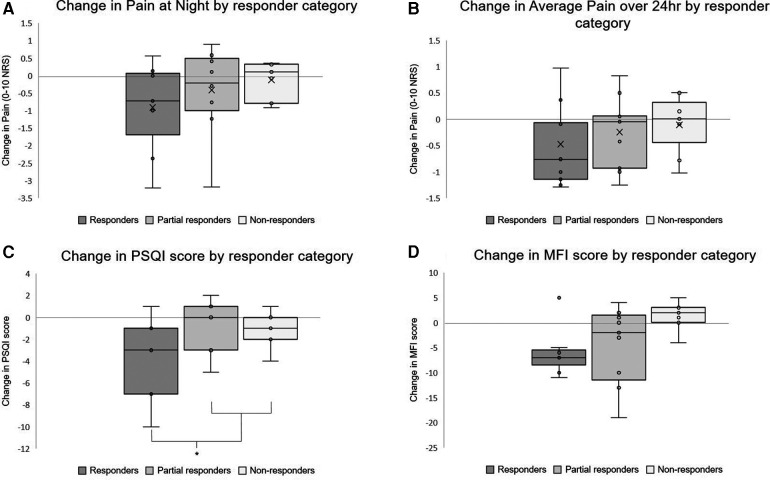
Boxplots showing selected outcomes by responder category. Responders are participants who reported an improvement in the brief pain inventory pain interference score by at least 1 point, partial responders by less than 1 point, non-responders showed no change or worsening. Arithmetic means represented by “X” in panels A and B, not included in the non-continuous variables. *Statistically significant difference between responders and other categories in PSQI change (*p* = 0.026).

Expanding on four selected variables of interest from Table 5, Figure 4 shows the response categories trending with other outcomes, as boxplots to visualise the spread of data. Panels A and B show change in daily diary measures of pain, at night and over 24 h respectively, during hBET use compared to baseline. Panel C shows greater improvements in sleep quality (measured with PSQI) in Responders (this group also being statistically significantly different to the other two groups) and boxplot D shows a trend for greater improvements in fatigue (measured with MFI) in Responders than in the other groups, although this was not statistically significant.

## 4. Discussion

Home-based, pre-sleep use of a smartphone programme for alpha entrainment by audio or visual stimulation was feasible for individuals with chronic pain and sleep disturbance. The findings are drawn from domiciliary use of hBET over 4 weeks, in a relatively unselected sample, which gives a level of ecological validity. This study represents the first application of alpha entrainment pre-sleep in those living with chronic pain and sleep disturbance, and the findings will inform the development of further trials in this area.

The hypothesised mechanism of action of hBET is a positive effect on both night time pain and improved sleep, subsequently leading to improved day time pain and related symptoms. Whilst it is well established that pain and sleep interact with each other, the purported inhibitory gating role of alpha could reasonably be expected to modulate both pain perception and sleep onset discretely, but the independent relevance of each in this population requires further study. Caution is needed in the interpretation of the changes in symptoms reported in this uncontrolled study, and conclusions about efficacy cannot be drawn. However, the experiences of users and pattern of response can usefully inform hypothesis generation and the design of future research. There is an indication that the improvements in patient-reported measures of sleep may be of larger magnitude or more readily observed than those of pain. Whilst the BPI pain severity score, which is derived entirely from 0 to 10 NRS pain ratings, did not improve across the whole group, the BPI pain interference score, which takes into account the impact of pain on various activities and functions, did improve. This may reflect different responsiveness of these measures over relatively short time periods in established chronic pain, or reflect the mechanism of action of this neuromodulatory intervention, and is not fully explained by this study. It is also notable that even the improved scores across many domains still remain above clinical thresholds, such as sleep efficiency (remains below 80%), sleep onset latency (remains over 30 min), anxiety and depression scores [remain over the “case” threshold of 8 (46)] and PSQI scores [remain markedly over the threshold of 6 indicating “poor sleepers”(40)]. The responder analysis is exploratory, and does not offer conclusions on effect, but adds weight to the notion that sleep and pain symptomology are acting together, and is consistent with the hypothesised mechanism that targeting both may lead to positively reinforcing benefits to daytime symptoms. It provides a possible approach to pre-specified response definition in future studies.

In this study, diary-reported measures of sleep onset, time and continuity improved whereas actigraphy assessed sleep measures were unchanged. Discrepancy between actigraphy and sleep diary, particularly in chronic pain, has been reported many times and given different interpretations. Systematic review and meta-analysis comparing methods of sleep assessment in chronic pain patients finds the most consistent discrepancy is that actigraphy gives lower estimates of Sleep Onset Latency than diary report, (by 23 min in meta-analysis) (47). This was the case in the current study, with overall 28 min difference found. This has often been referred to as sleep misperception, with the inference that actigraphy provides a superior “objective” measure. Noting that daytime experience plays a large part in how people with chronic pain judge their sleep (48) one interpretation is that sleep diary and actigraphy are measuring different constructs. However, it is also possible that actigraphy may be less accurate in this group. Studies using polysomnography have contested the idea that sleep complaints in fibromyalgia are due to sleep misperception (49) and when systematically reviewed, actigraphy is found to overestimate total sleep time and underestimates sleep onset latency and wake after sleep onset time, compared to polysomnography, in adults with chronic conditions (50). Future studies incorporating electroencephalographic (EEG) monitoring would provide the multiple benefits of more accurate and detailed sleep determination and staging, insight to the validity of actigraphy in this population and direct evaluation of the alpha entrainment effect of this intervention. It is also possible that EEG monitoring could optimise the intervention, either through personalisation to the individual peak alpha, or by using closed-loop stimulation.

Limitations of this study mean it is not possible to conclude that the observed improvements result only from the intervention, as this is an uncontrolled, open-label feasibility study, not designed to determine efficacy. The limitations include the possibility of bias resulting from participant or researcher enthusiasm for the open-label intervention, placebo and secular (time) effects. Participants were aware of the aim of the study and that they were trialling a novel approach to chronic pain which may have appealed and promoted a placebo effect, which is not controlled for. This was mitigated against with transparency from the researchers and study documentation that the effectiveness of the intervention is as yet unknown. A passive effect due to time, such as regression to the mean, is unlikely as the participants had very longstanding symptoms of median 9 years and were not recruited in a way which would enrich the sample with those experiencing acute “flares” of symptoms. Bias in participant reported outcomes was mitigated against by encouraging honest responses. Diary entries may be less susceptible to this bias, being completed iteratively day after day, and it is notable that the findings from diary entries agree with those from baseline and completion questionnaires, although they are generally of smaller effect size. Gender and age were not controlled for at this feasibility stage, but are likely to be relevant covariates in the effect on symptoms, which should be accounted for in future study design. Finally, the study only considers a relatively short time period of four weeks of intervention use, with no longer term follow up. This leaves open the possibility of either a novelty effect exaggerating the benefits, or a more incremental effect being missed due to the short duration of this study. Anecdotal clinical observations tend to suggest that sleep improvements precede improvements in pain, which would require longer a study duration to capture.

In conclusion, hBET appears to be a feasible intervention for the home setting and evaluation under controlled conditions of its clinical effect is warranted.

## Data Availability

The raw data supporting the conclusions of this article will be made available by the authors, without undue reservation.
