# Supplementary material for: A feasibility study of pre-sleep audio and visual alpha brain entrainment for people with chronic pain and sleep disturbance
Source: Front Pain Res (Lausanne). 2023 Feb 23;4:1096084. doi: 10.3389/fpain.2023.1096084 (PMC9996154; doi:10.3389/fpain.2023.1096084)
Supplement: Supplementary file 1 [file Table1.docx]

**Supplementary material: Audio and Visual hBET modalities disaggregated**

**Table S1.** Sleep and pain diary results for Audio hBET (n=23)

|  | Mean (SD) at Baseline (161 nights) | Mean (SD) in Audio hBET condition (458 nights) | Change (Audio compared to baseline) | P value for paired difference* between audio and baseline | Effect size |
| --- | --- | --- | --- | --- | --- |
| Average pain over 24 hours (0-10 NRS) | 6.5 (1.9) | 6.3 (1.8) | -0.2 | 0.0949 |  |
| Average pain at night (0-10 NRS) | 6.0 (1.9) | 5.5 (2.1) | -0.5 | **0.0441** | 0.45** |
|  |  |  |  |  |  |
| Sleep Onset Latency (mins) | 51.1 (39.7) | 38.7 (27.0) | -12.3 | **0.0056** | 0.58*** |
| Wake After Sleep Onset (mins) | 38.0 (32.9) | 28.7 (26.6) | -9.3 | **0.0459** | 0.42*** |
| Total Sleep Time (mins) | 390.3 (82.1) | 414.5 (87.3) | 24.2 | **0.0234** | 0.51** |
| Sleep efficiency (%) | 74.4 (11.3) | 78.8 (11.2) | 4.4 | **0.0007** | 0.82** |
|  |  |  |  |  |  |
| Median quality rating (0-5 scale) | 2.4 (1.0) | 2.4 (1.0) | 0.0 | 1 |  |
| Median refreshed rating (0-5 scale) | 1.5 (0.9) | 2.2 (1.1) | 0.6 | **0.0018** | 0.7*** |
| Median number of awakenings | 3.1 (2.3) | 2.3 (2.1) | -0.8 | **0.0108** | 0.5*** |
|  |  |  |  |  |  |
| % nights with quality rated 3+ | 52.2 (30.6) | 55.4 (32.2) | 3.2 | 0.6115 |  |
| % mornings with refreshed rated 3+ | 26.6 (23.0) | 43.7 (32.3) | 17.1 | 0.2173 |  |

*Paired t-test for continuous and normally distributed variables, Wilcoxon sign rank test for non-continuous or non-normally distributed variables

**Cohen's d for t-tests

***r for Wilcoxon sign rank tests

**Table S2.** Sleep and pain diary results for Visual hBET (n=13)

|  | Mean (SD) at Baseline (91 nights) | Mean (SD) in Visual hBET condition (175 nights) | Change (Visual compared to baseline) | P value for paired difference* between Visual and baseline | Effect size |
| --- | --- | --- | --- | --- | --- |
| Average pain over 24 hours (0-10 NRS) | 6.1 (2.1) | 5.5 (1.7) | -0.6 | 0.0884 |  |
| Average pain at night (0-10 NRS) | 5.6 (2.1) | 5.3 (2.0) | -0.3 | 0.2909 |  |
|  |  |  |  |  |  |
| Sleep Onset Latency (mins) | 56.6 (51.0) | 37.2 (33.7) | -19.4 | **0.0418** | 0.64** |
| Wake After Sleep Onset (mins) | 23.3 (27.1) | 17.4 (27.5) | -5.9 | 0.2783 |  |
| Total Sleep Time (mins) | 366.1 (81.3) | 408.1 (97.9) | 42.0 | **0.0303** | 0.69** |
| Sleep efficiency (%) | 76.9 (12.0) | 81.8 (11.4) | 4.8 | 0.0581 |  |
|  |  |  |  |  |  |
| Median quality rating (0-5 scale) | 2.9 (0.6) | 3.0 (0.4) | 0.1 | 0.7127 |  |
| Median refreshed rating (0-5 scale) | 1.5 (0.8) | 2.7 (0.6) | 1.1 | **0.0022** | 0.85*** |
| Median number of awakenings | 2.9 (2.8) | 2.3 (2.9) | -0.6 | **0.0473** | 0.55*** |
|  |  |  |  |  |  |
| % nights with quality rated 3+ | 62.6 (27.7) | 72.2 (16.2) | 9.5 | 0.2983 |  |
| % mornings with refreshed rated 3+ | 27.5 (17.0) | 55.3 (23.3) | 27.8 | **0.0042** | 0.79*** |

*Paired t-test for continuous and normally distributed variables, Wilcoxon sign rank test for non-continuous or non-normally distributed variables

**Cohen's d for t-tests

***r for Wilcoxon sign rank tests
